# Impact of viral features, host jumps and phylogeography on the rapid evolution of Aleutian mink disease virus (AMDV)

**DOI:** 10.1038/s41598-021-96025-z

**Published:** 2021-08-12

**Authors:** Giovanni Franzo, Matteo Legnardi, Laura Grassi, Giorgia Dotto, Michele Drigo, Mattia Cecchinato, Claudia Maria Tucciarone

**Affiliations:** Department of Animal Medicine, Production and Health (MAPS), University of Padua, 35020 Legnaro, PD Italy

**Keywords:** Molecular evolution, Phylogenetics, Microbiology, Microbial genetics, Virology

## Abstract

Aleutian mink disease virus (AMDV) is one the most relevant pathogens of domestic mink, where it can cause significant economic losses, and wild species, which are considered a threat to mink farms. Despite their relevance, many aspects of the origin, evolution, and geographic and host spreading patterns of AMDV have never been investigated on a global scale using a comprehensive biostatistical approach. The present study, benefitting from a large dataset of sequences collected worldwide and several phylodynamic-based approaches, demonstrates the ancient origin of AMDV and its broad, unconstrained circulation from the initial intercontinental spread to the massive among-country circulation, especially within Europe, combined with local persistence and evolution. Clear expansion of the viral population size occurred over time until more effective control measures started to be applied. The role of frequent changes in epidemiological niches, including different hosts, in driving the high nucleotide and amino acid evolutionary rates was also explored by comparing the strengths of selective pressures acting on different populations. The obtained results suggest that the viral passage among locations and between wild and domesticated animals poses a double threat to farm profitability and animal welfare and health, which is particularly relevant for endangered species. Therefore, further efforts must be made to limit viral circulation and to refine our knowledge of factors enhancing AMDV spread, particularly at the wild-domestic interface.

## Introduction

Aleutian disease is probably the most relevant cause of economic losses in global mink farming, in terms of both direct and control-associated costs^1^. The disease is caused by a virus classified as the species *Carnivore amdoparvovirus 1* in genus *Amdoparvovirus* and typically features a progressive immune-complex-related syndrome leading to glomerulonephritis and arteritis, although the clinical outcome is affected by the interactions between Aleutian mink disease virus (AMDV) strain virulence and host-related factors^2,3^. An acute syndrome can also occur in newborn minks and is characterized by often fatal interstitial pneumonia^4^. Due to the North American origin of the farmed mink species, AMDV was thought to have originated from this area before or after domestication, later spreading to other countries because of local and international animal trades^5^. However, definitive and robust evidence on this topic is not available, and the origin of AMDV from another area and/or feral mustelid species cannot be excluded^6^.

Similar to other members of the *Parvoviridae* family, AMDV is characterized by single-stranded DNA (ssDNA) ∼ 4.8 kb in size encoding 3 nonstructural (NS1-3) and 2 capsid structural proteins (VP1-2)^7^. In most viruses, higher phenotypic variability and genotypic variability affect structural proteins that evolve under the pressure of escaping the host immune response. However, likely because of the antibody-mediated enhancement that takes part in the viral infectious cycle, such pressure is not present. The primary replication site for AMDV is in circulating macrophages, and viral entry is mediated by cellular Fc receptors recognizing antibody-coated viral particles^8^. Therefore, escape from the host immune response could paradoxically be detrimental to viral fitness^9^. On the other hand, NS proteins, particularly NS1, which is essential for viral replication, are characterized by marked genetic variability and have thus been widely studied and sequenced for epidemiological purposes^5,10^, allowing the identification of different clusters. The causes of NS protein variability are still uncertain, although a combination of the high evolutionary rate typical of ssDNA viruses and immunity-induced selective pressures can be hypothesized. Except for local aggregates, clear geographical grouping does not seem to occur^11^. Several small-scale studies reported noteworthy diversity in the same region or even within farms, suggesting that long- and short-distance animal trades are likely to play a relevant role in shaping the wide and largely unconstrained viral circulation of AMDV and its epidemiological patterns^5,12^.

In addition to American minks (*Neovison vison*), AMDV is known to infect other mustelids, including European minks (*Mustela lutreola*), ferrets (*Mustela putorius furo*), short-tailed weasels (*Mustela erminea*), European pine martens (*Martes martes*), stone martens (*Martes foina*), European otters (*Lutra lutra*), North American river otters (*Lontra canadensis*) and Eurasian badgers (*Meles meles*), and carnivorous species, including striped skunks (*Mephitis mephitis*), raccoons (*Procyon lotor*), bobcats (*Lynx rufus*) and common genets (*Genetta genetta*)^11^.

The scenario is further complicated by the presence of feral minks, which have become established across Europe and America as a result of escape and deliberate releases by animal rights activists^5^. Additionally, in this case, whether AMDV is native to wild minks and was transmitted to captive mink populations or the virus originated in mink farms and wild animals were infected through accidental escapes or deliberate releases of infected captive animals is still a matter of discussion^5^. The respective roles of wild and domestic populations in infection maintenance and the prevalent direction of viral flux are similarly unknown.

Finally, the features of wild and domestic populations (e.g., effective contacts, population size and density, turnover, immunity, health status, and genetic background) could affect both viral population size and selective pressures acting on the pathogen, thus differentially contributing to AMDV evolution and new variant emergence.

The aim of the present work is to provide a comprehensive depiction of epidemiological AMDV patterns at the global scale and investigate how viral mutation, population size, the interaction with the host and geographical spreading have shaped AMDV evolution over time at the genomic level, using different biostatistic and phylodynamic approaches.

## Results

### Datasets

Based on the selected inclusion criteria and after the removal of poor-quality sequences (i.e., poorly aligned ones and those with frameshift mutations or premature stop codons), a dataset of partial NS1 (286 bases long) sequences, including 927 sequences with known collection dates (spanning the period 1963–2019) and countries (19 countries) and 689 sequences with known collection dates and hosts (5 hosts), was obtained. In the latter case, the status of the mink host (farmed or wild) was available for 623 out of 675 strains. The list of used sequences is provided in the Appendix.

Additionally, 94 complete NS1 sequences with known sampling hosts were included in the study. The phylogenetic signal was adequate for all datasets, including the randomly generated ones. The temporal signal, evaluated using the same databases, demonstrated a constantly positive correlation between root-to-tip distance and collection date, ranging between 0.27 and 0.38, and the estimated evolutionary rate was approximately 10^–4^ substitutions/site/year. Therefore, despite not being strong, the temporal signal was present and considered adequate for further analysis.

### Viral population parameters and phylogeographic analysis

**Figure 1:**
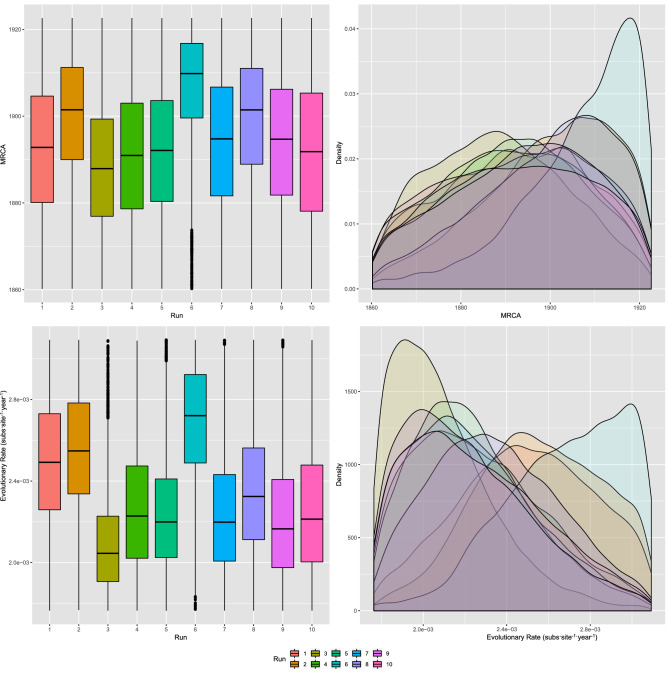
AMDV MRCA and evolutionary rate. Upper figure: Boxplot (left) and Densityplot (right) of the MRCA posterior probability. Lower figure: Boxplot (left) and Densityplot (right) of the mean evolutionary rate (expressed in base-10 logarithm) posterior probability. Results have been estimated performing ten independent runs based on randomly sampled sequences. The 95HPD intervals are reported for both figures.

**Figure 2:**
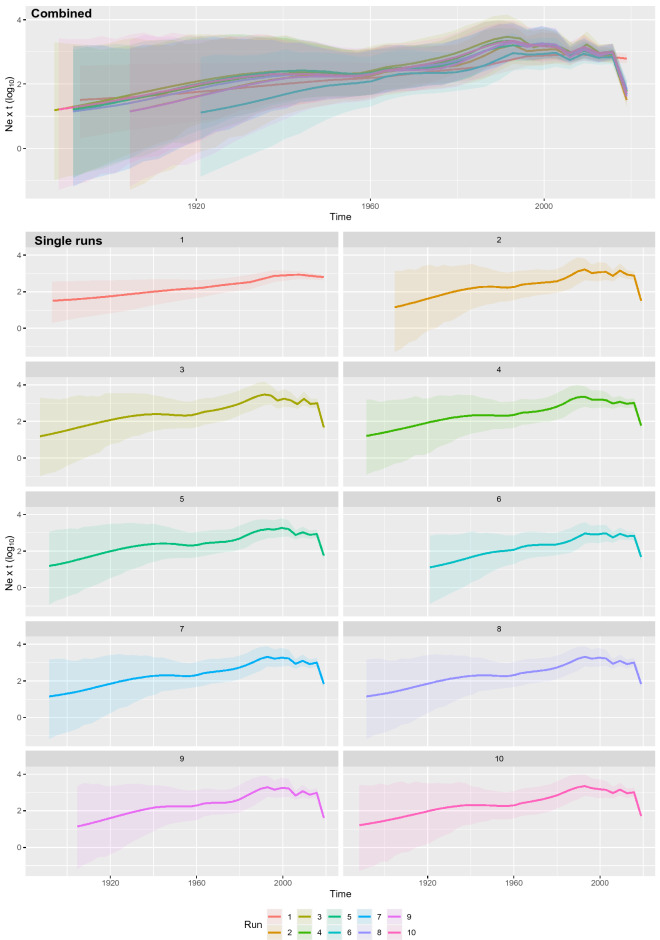
AMDV population dynamics. Mean relative genetic diversity over time estimated performing ten independent runs (colour-coded) based on randomly sampled sequences. Results have been reported both superimposed (upper figure) and individually (lower figures) for comparison purpose. The lines represent the mean value while the shaded areas depict the 95HPD ranges.

The AMDV MRCA was estimated to 1885.57 (median = 1895.32; 95 HPD = 1799.12–1933.67). Fully comparable values were obtained based on the ten randomly generated datasets (Fig. 1). Similarly, the overall analysis and the analyses based on random databases produced analogous evolutionary rate estimates (mean = 2.23 × 10^–3^; median = 2.23 × 10^–3^; 95 HPD = 1.4 × 10^–3^–3.7 × 10^–3^) (Fig. 1). Marginal likelihood estimation suggested a relaxed molecular clock rather than a strict clock. The analysis of viral population dynamics revealed a consistent pattern, independent of the randomly included sequences, featuring a progressive increase in viral relative genetic diversity from the AMDV origin to approximately the 1990s, when a stationary phase began, followed by a slow decline at the beginning of the new millennium and a more abrupt decline in the last years of the study (Fig. 2).

**Figure 3:**
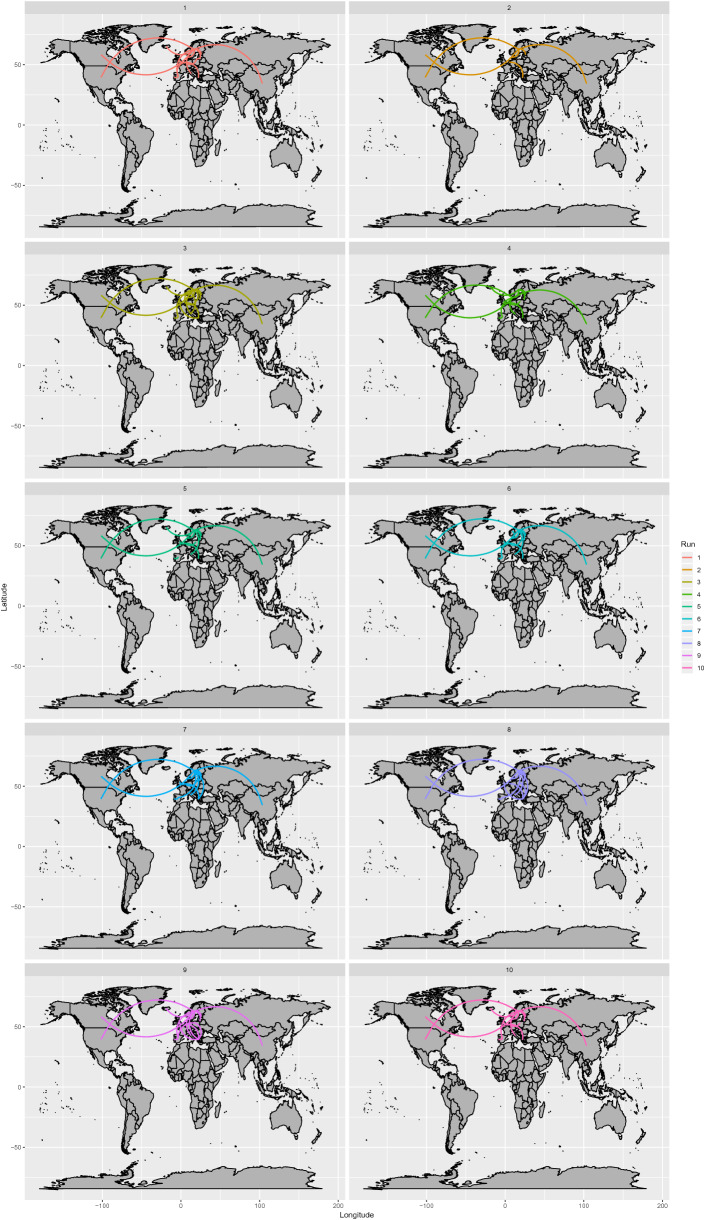
AMDV migration paths. Well supported migration paths (i.e. BF > 10) among countries are depicted. Different frames report the results of independent runs. Maps were generated using the ggplot^54^ library in R.

The phylogeographic model evaluation did not support an asymmetric migration model over the symmetric model based on Bayesian Factor (BF) calculation, independent of the considered random dataset. Despite a certain degree of variability among runs in the among-country analysis of inferred significant migration rates, some consistent features could be identified (Fig. 3 and Supplementary Figure 1). With the exception of only Run 1, where the AMDV origin was estimated to be in North America, in all instances, an initial European source was predicted, involving Northern European countries in particular. Nevertheless, the posterior probability associated with the country of origin was constantly low (essentially between 0.5 and 0.8) (Supplementary Figure 2), demonstrating the uncertainty of this prediction. Several well-supported (BF > 10) migration rates were detected between countries and involved long-distance connections between North America and Europe and between Europe and China, as well as several within-Europe links. In all runs, independent connections of Europe with the USA and Canada were identified, linked particularly to Sweden, from which many additional significant migration paths with other European countries were proven, although a certain degree of variability in European connections occurred depending on the specific dataset (Fig. 3).

The overall geographic pattern featured different clusters of strains collected from the same country (Supplementary Figure 1), which is suggestive of multiple strain introductions in the same area, followed by local persistence, evolution and co-circulation.

### Structured coalescent

The application of a structured coalescent model, considering the hosts of origin as different *demes*, provided concordant estimates compared to those of the previously described serial coalescent method in terms of tMRCA and evolutionary rates. The host of origin was predicted to be “Other wild animals” with a high posterior probability (i.e., > 0.9) (Supplementary Figure 3). Thereafter, several host switches/jumps were inferred, particularly between domestic minks and wild animals. More specifically, the estimated migration rates were as follows: farmed mink to wild mink = 2.88 × 10^–2^, farmed mink to other wild animals = 1.71 × 10^–2^, wild mink to other wild animals = 1.48 × 10^–3^, other wild animals to farmed mink = 1.54 × 10^–2^, other wild animals to farmed mink = 1.75 × 10^–3^ and other wild animals to wild mink = 2.44 × 10^–3^.

### Selective pressure analysis

**Figure 4:**
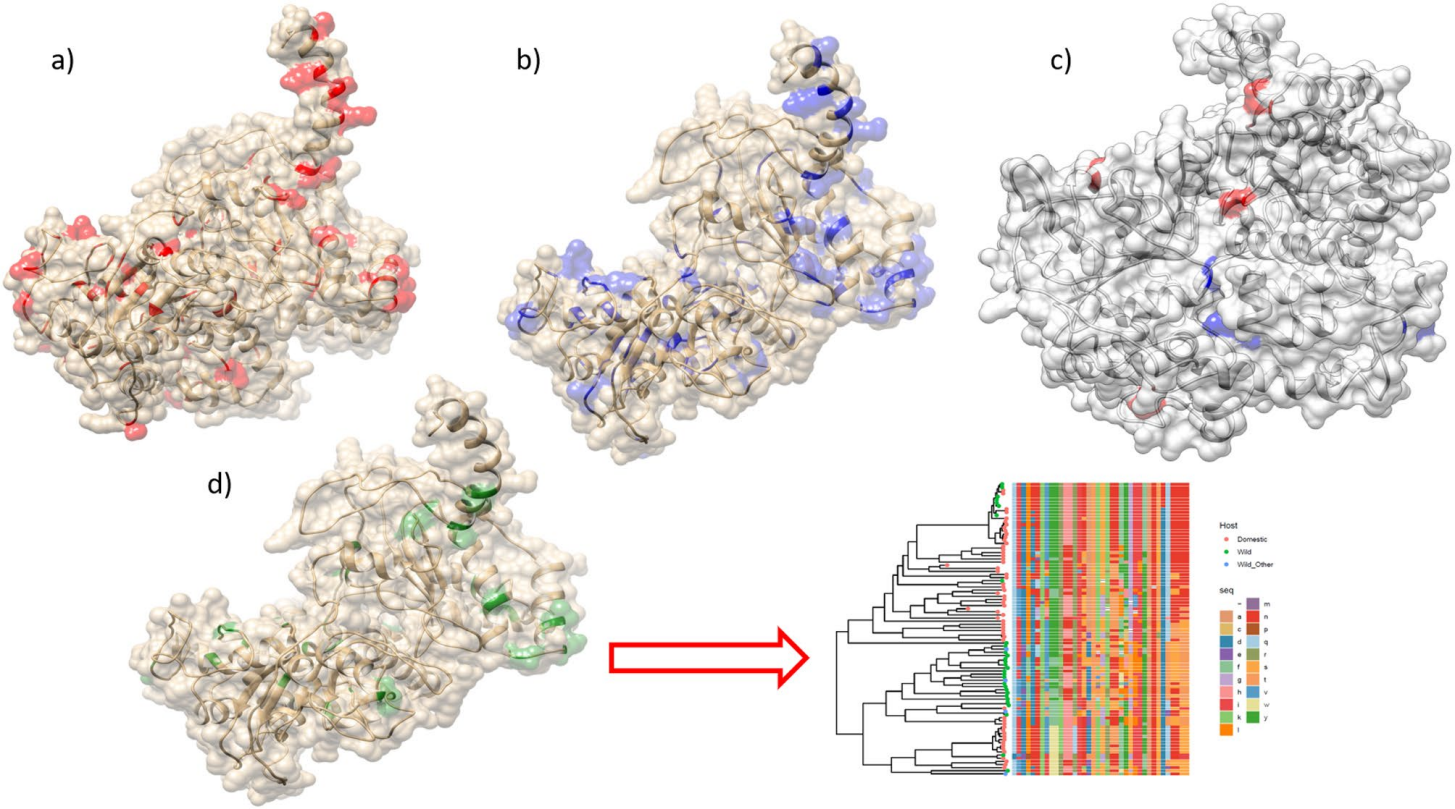
Tertiary structure of the NS1 protein estimated through homology modelling. Figures were generated using the Chimera software^53^. Amino acids ribbon and relative surface (when exposed) have been colour-coded to depict the results of different selective pressures analyses: (**a**) sites detected under pervasive diversifying selection are highlighted in red; (**b**) sites detected under episodic diversifying selection are highlighted in blue; (**c**) sites detected under a significantly higher diversifying selection in domestic and wild minks are highlighted in red and blue, respectively; (**d**) (left figure) sites detected under episodic directional selection (wild subjects collected sequences have been used as foreground), (right figure) plot reporting the alignment of the NS1 protein amino acids under episodic directional selection with respect to their position in the phylogenetic tree (strains have been colour-coded according to the collection host with the highest posterior probability). To facilitate the evaluation of specific patterns, the latter plot is provided as Supplementary Figure 5 also.

The analysis of pervasive selective pressure revealed the presence of different sites under diversifying selection detected as significant by at least two implemented methods (Supplementary Figure 4 and Supplementary Table 1). Similarly, several codons were demonstrated to be under episodic diversifying selection by MEME (Supplementary Table 2). In both instances, homology modelling of the NS1 protein allowed the location of sites under diversifying selection on the protein surface (Fig. 4).

The comparison of selective pressure patterns acting on the strains collected from farmed and wild/feral minks demonstrated a significant difference in the proportion of sites under selection (p < 0.001) but not in the overall selection strength (p = 0.854) or selective regime (p = 0.853). Accordingly, the site-by-site comparison performed on the alignment of strains collected from domestic and wild/feral minks revealed 12 sites (i.e., codons 12, 27, 83, 120, 153, 211, 317, 345, 353, 393, 450 and 484) with differential selective pressure and exposed on the NS1 surface (Fig. 4). For 8 of these codons, i.e., 16, 27, 83, 120, 211, 353, 393 and 484, the dN/dS ratio was higher in domestic animals. Finally, DEPS analysis highlighted 33 sites under episodic directional selection between wild and domestic minks (Supplementary Table 3). Additionally, in this case, the involved amino acids were located on the predicted surface of the NS1 protein (Fig. 4).

## Discussion

AMDV is one of the most relevant pathogens of minks and mustelids worldwide. It can cause severe economic losses in commercially raised minks, especially since no vaccine is currently available and control must be based on eradication programs^13,14^. However, viral features, including persistent infection and environmental resistance^2,15^, make this approach challenging. The risk of new introductions from other farms or even wild animals further complicates the scenario. Conversely, viral spread from domestic to wild animals can pose a threat to these species, particularly endangered ones^5^. Despite their relevance, many of the aspects of AMDV epidemiology have not been quantitatively evaluated on a global scale. In contrast to several previous studies limited to single countries, locations or even individual farms^6,15,16^, the present study attempts to complement their results by providing an overall depiction of AMDV molecular epidemiology and evolution from the AMDV origin to the present, benefitting from the coalescent theory robust framework, which allowed us to statistically support the performed analysis rather than rely on subjective evaluation of strain genetic distances and/or phylogenetic analysis. The viral MRCA was estimated to the end of the XIX century. Although previous studies have reported the absence of temporal signal in AMDV evolution^6^, when preliminarily evaluated using randomly generated datasets, a positive correlation was constantly detected between sampling date and root-to-tip distance, which suggests a certain temporal structure. Additionally, the relaxed molecular clock model was preferred over the strict model based on BF calculations. It was thus possible to conclude that AMDV evolution follows a temporal structure, compliant with a molecular-clock evolutionary model, although with significant variations over time and/or among lineages. Based on these considerations and the high repeatability of the results obtained using the randomly generated datasets and different analytical models, we considered our AMDV origin estimates reliable. It can therefore be concluded that AMDV originated well before its first identification as a clinical problem in fur minks. The actual host of origin is still largely unknown and a matter of discussion, with transmission from wild minks to domestic minks or domestic emergence and subsequent “escape” to the wild being the two debated scenarios^5,6,17^. The present study estimation reveals a high probability of a wild origin, albeit in non-mink species. While unexpected, the scenario could be supported by the markedly high AMDV circulation in several wild species^17^. Mink farming began in the late XIX century in North America^5^, approximately in the upper range of the tMRCA estimated by our analyses. Initial breeding stocks were acquired from locally caught wild minks; thus, clear compartmentalization between wild and domestic animals did not occur in the early phases, likely facilitating strain exchange in a “homogeneous” and connected wild/domestic environment. This environment, at the same time, might have created favourable circumstances for viral expansion due to nascent farming conditions. The viral population size showed a progressive increase over time until recent decades, which conforms to the proposed scenario. The subsequent stabilization and decrease in viral expansion could be attributed to the implementation of more effective control measures. For example, AMDV in Denmark has been subjected to a control campaign since 1976, but eradication has been supported by legislation since 1999^13^, when an actual effect on AMDV relative genetic diversity became clear (Fig. 2). The heterogeneity of control programmes around the world and continuous viral circulation in feral minks and wild animals can, on the other hand, explain the limited overall reduction compared to expectations based on Danish epidemiological data.

Of note, AMDV demonstrated a high evolutionary rate, in the upper range typical of ssDNA viruses^18^. From this perspective, AMDV appears more similar to other parvoviruses that underwent a host jump, such as canine parvovirus, than to others, such as feline parvovirus, that are well adapted to a specific host^19,20^. This evidence could indicate progressive adaptation to new hosts or at least ecological niches, which could be consistent with our non-mink origin hypothesis.

However, other non-conflicting processes could have at least partially contributed to the observed high evolutionary rate, including the host population size, density in the farming setting and the enhanced transmission rate.

The analysis of selective pressures demonstrated the presence of a significant number of NS1 protein amino acids under pervasive and, even more, episodic diversifying selection, in agreement with the findings of other studies^10,21^.

Homology modelling revealed that these amino acids were mostly exposed on the protein surface. Even if the absence of an experimentally determined tertiary structure demands caution, the observed scenario suggests a relevant role of immune-induced pressure in shaping NS1 evolution. Interestingly, the comparison of strains collected from wild and domestic animals yielded a significant difference in the selective force distribution, and the dN/dS ratio was proven to be different at several codon positions, being higher in domestic animals in 8 out of 12 instances. Evidence of directional selective pressures was also uncovered. Taken together, these results could be indicative of adaptation to new environmental conditions. Of note, also in these cases, the involved sites were exposed on the NS1 surface, which could reflect the need for a differential interaction with host proteins and/or the immune system. Variations in population immunity ascribable to differences in prevalence, host genetic background or immune system functionality (pathophysiologic) might be only some of the potentially involved factors.

However, the limited number of available complete NS1 sequences, especially from wild animals, does not allow us to confidently exclude the presence of a “founder” effect (i.e., when foreground and background strains in the phylogenetic tree (see “Material and methods”) are separated by a single or few branches) rather than actual directional selection^22^. In fact, when such a scenario occurs, it is almost impossible to discriminate whether the observed amino acid profiles are due to random and casual selection on an ancestor carrying a certain phenotype or to causal forces favouring the specific amino acid variant. Further investigations would help solve this issue when more sequences are available.

Nevertheless, after the revelation of a likely wild origin, the analysis of viral flux highlighted a preeminent directionality from domestic to wild populations, which fits well with the frequent introduction of domestic animals to the wild due to escapes or deliberate releases by animal rights movements^23–25^. The presence of large wild-only clusters (Supplementary Figure 3) confirms the capacity of AMDV to persist and evolve over time in this environment independently of new introductions^11^. Wild species should thus be considered more threatened by domestic species rather than a threat to those species. Nevertheless, as the viral migration rate from wild to farmed animals is not zero, it is clear that biosecurity measure improvement is still warranted and could contribute to the prevention of new outbreaks^5^.

Nevertheless, the present study and other studies demonstrated that the main risk is represented by the introduction of new strains from other farms^13^. An intense transmission network was reconstructed over long distances (i.e., different countries and continents), implying artificial movements of animals or other contamination sources such as fomites^26^.

In particular, the link between North America and Northern European countries has already been proposed^1,5^. Our estimates support a European origin, which poorly fits with historic epidemiological data: the = mink was initially domesticated in North America and thereafter extensively exported to Northern Europe^27^. Therefore, a North American origin of AMDV, followed by exportation to Europe, would appear more likely. The limited number of old sequences jeopardizes the ability to estimate such old spreading events. Long branches and the lack of historical data largely prevent inference of the spatial history of older viral lineages with high confidence. Additional spatial movements between multiple locations are likely concealed in the “long tree branches” during this time frame^28^. Accordingly, also from a statistical perspective, the symmetric migration model was preferred over the asymmetric model, highlighting the lack of evidence supporting significant directionality of the viral flux between country pairs. Above all, the disproportion of sequences collected from Europe could have biased the results, despite our attempts to create more balanced datasets by random sampling. Therefore, based on these considerations and with caution, migration patterns should be evaluated in terms of ‘‘contact” among countries, avoiding overinterpretation of their directionality.

European countries were also involved in most of the mink trades and AMDV spreading events, as previously suggested by other authors^6,11,15^. Interestingly, the mink farms in some of these countries were fully integrated into the Soviet Union’s mink-farming industry before independence, which could have contributed to eastward viral dispersal^11^. In China, mink farming started in the 1950s, likely with imports from the Soviet Union^29^, which fits with our estimate of AMDV introduction to this country in the late 1950s. Unfortunately, Asian sequences with adequate features for inclusion in our dataset were essentially limited to China, preventing higher resolution of the viral spread to and among oriental countries. In contrast, the abundance of European sequences allowed us to demonstrate an intense diffusion of AMDV within this area. Frequent introduction events in different countries were identified and followed by local persistence and evolution, which suggests the limited efficacy of biosecurity measures both among and within countries, similar to what has been described for several other livestock infections^30–32^. Likely, a comparable scenario could be true for other regions, including Asia.

The present study provides several details regarding AMDV epidemiology and evolution, which appear to occur in multiple environments linked by sporadic contact. AMDV seems to persist and evolve in different wild or domestic hosts, even for a noteworthy time, sporadically jumping to other hosts and beginning a new pathway^11^. Similarly, despite frequent and multiple introductions to new geographic areas that characterize AMDV epidemiology, local persistence and evolution also occur. These conditions likely explain some of the peculiarities of the viral strains, as well as the high but heterogeneous evolutionary rate of AMDV, which periodically switches between stable and variable environmental conditions. From a practical perspective, the limited efficacy of currently applied control measures in preventing viral passage among countries and farms and contact between wild and domesticated animals were also demonstrated. Such conditions pose a double threat to farm profitability along with animal welfare and health, which is particularly relevant for endangered species. Further efforts must thus be made to limit such viral spreading, and dedicated studies should be performed to refine our knowledge of AMDV spreading risk/enhancing factors, particularly at the wild-domestic interface.

## Material and methods

### Sequence dataset preparation

Freely available NS1 sequences were considered in the present study, aiming to reconstruct AMDV population dynamics, phylogeography and among-host transmission.

Only a limited number of complete NS1 sequences, properly annotated with metadata of interest (i.e. collection date, sampling host and country) were available. To deal with this data shortage and maximize available information, different datasets were created according to the main purpose of the specific analysis, and the partial NS1 region with the highest coverage of available sequences was selected.

Therefore, only sequences with available collection date were selected to estimate evolution rate and population dynamics. Similarly, phylogeographic and host transmission analyses were performed separately on sequences with known sampling date, collection country and host, respectively.

For each database, recombinant sequences were identified using RDP4^33^ and excluded from further analysis. The RDP4 settings for each method were adjusted to account for the dataset features according to the RDP manual recommendations. In particular, RDP, GENECONV, Chimaera and 3Seq were used in a primary scan while the full set of available methods was used for the analysis refinement. Only recombination events detected by more than 2 methods with a significance value lower than 10^–5^ (p-value < 10^–5^) and Bonferroni correction were accepted. The absence of residual recombination signal was assessed by GARD^34^. The presence of an adequate phylogenetic signal despite the relatively short selected sequence region was assessed using the likelihood mapping approach implemented in IqTree^35^. A maximum likelihood (ML) phylogenetic tree was reconstructed using the same software selecting as substitution model the one with the lowest Akaike information criterion (AIC) and used to preliminary evaluate the temporal signal using TempEst^36^.

A dataset of complete NS1 sequences was also downloaded and processed as previously described.

### Sequence analysis

#### Viral population parameters and phylogeographic analysis

The evolutionary rate, time to the most recent common ancestor (tMRCA) and population dynamics were jointly estimated in a Bayesian fashion using the serial coalescent approach in BEAST 1.8.4^37^. The best substitution model (i.e. GTR + G) was selected based on the Bayesian Information Criterion (BIC) calculated using Jmodeltest 2^38^, while the relaxed lognormal molecular clock^39^ was preferred over the strict one based on the BF value, calculated through the marginal likelihood estimation using the Path Sampling and Stepping Stone methods, as described by Baele et al.^40^.

The non-parametric skygrid^41^ model was selected to account for and reconstruct the viral population dynamics.

A comparable approach was implemented on the dataset of the sequences with available collection country to reconstruct the viral spreading over time using the discrete state phylogeographic approach described by Lemey et al.^42^. The symmetric migration rate was preferred over the asymmetric one based on the marginal likelihood calculation of the two models^40^. The Bayesian Stochastic Search Variable Selection (BSSVS) was also implemented, allowing the definition of the most parsimonious viral dispersal path and the identification of the statistically supported migration rates between countries through BF testing^42^. The sequence availability differed significantly among countries and time periods, potentially biasing the analysis results. For this reason, attempting to control and limit this phenomenon, 10 more balanced dataset were created by randomly selecting without replacement up to a maximum of 10 sequences for each country-year pair.

For each dataset, 200 million generations-long runs were performed, sampling trees and parameters every 20 thousand generations. Run results were accepted only if Estimate Sample Size (ESS) exceeded 200 and mixing and convergence, evaluated by visually inspecting the trace plots using Tracer 1.6, were adequate. Population parameters were summarized as mean and 95% Highest Posterior Density (95HPD) after discarding the first 20% of the run as burn-in. The annotated maximum clade credibility tree was generated using Treeannotator, after discarding the first 20% of the posterior trees.

Overall, a BF > 10 was considered adequate to prefer the more complex model over the simpler ones. Similarly, migration rates among countries were considered non-zero (i.e. well supported) when the BF, calculated using SPREAD3^43^ was greater than 10.

### Structured coalescent

The connections and viral flux featuring wild and domestic animals were evaluated using a structured coalescent analysis. Viral strains were categorized as originating from “Farmed mink”, “Wild mink” and “Other wild animals” based on information present in Genbank or literature search.

Briefly, according to structured coalescent, each of these groups was treated as a separate *deme* with a certain size, connected to other *demes* through strain migration occurring at a certain rate. The advantage of this model over discrete trait analysis is that migration events affecting lineages are explicitly parameterized and estimated, avoiding the potentially biasing assumption that sampling intensity is proportional to subpopulation size^44,45^. Such an assumption can be particularly strong when dealing with wild animals, whose sampling is especially challenging and economical relevance is typically marginal, further reducing the related research activities.

The partial NS1 sequences, for which the collection host category was available were analyzed using the structured coalescent approach as implemented in the package BASTA^45^ of BEAST2.5^46^. The substitution and clock models were respectively selected based on the BIC and BF values (calculated through estimation of the marginal likelihood using the Path Sampling and Stepping Stone methods). A MCMC run of 200 million generations was performed sampling trees and parameters every 20 thousand generations. Run results were accepted only if ESS exceeded 200 and mixing and convergence, evaluated by visually inspecting the trace plots using Tracer 1.6, were adequate. Population parameters were summarized as mean and 95HPD after discarding the first 20% of the run as burn-in. The annotated maximum clade credibility tree was generated using Treeannotator, after discarding the first 20% of the posterior trees.

### Selective pressure analysis

The dataset of complete NS1 sequences was used to estimate sites under different evolution pressures applying different methods, all based on the comparison of relative rates of non-synonymous (dN) to synonymous (dS) substitutions. The SLAC, FEL and FUBAR methods^47,48^ were selected to estimate codons under pervasive purifying or diversifying selection, while the MEME method^49^ was used to evaluate the occurrence of episodic diversifying selection. All methods are implemented in the HYPHY software^50^. The significance level was set to p < 0.05 for SLAC, FEL and MEME, and to a posterior probability higher than 0.9 for FUBAR.

The comparison of selective forces acting on wild and domestic animals was performed with different statistical approaches. The presence of an overall different selection strength, proportion of sites under selection and selective regime (both strength and proportion) between alignments of strains collected from farmed and wild minks was assessed using the *dNdSDistributionComparison.bf* function of the HYPHY package. Differences in the site by site-selective pressure strength between the two alignments were also evaluated using the *CompareSelectivePressure.bf* function of the same package. Finally, the occurrence of episodic directional selection was tested with MEDS^51^, considering phylogenetic tree branches leading to strains collected from wild-animals as foreground.

To tentatively evaluate the spatial distribution of domains under selective pressures, the tertiary structure of the NS1 was estimated through homology modelling, using Phyre2^52^. The obtained model was edited and visualized using Chimera^53^.

## Supplementary Information

Supplementary Information 1.

Supplementary Information 2.

Supplementary Information 3.

## Data Availability

Accession number and relevant metadata of uses sequences are available in Appendix.
