# Supplementary material for: Impact of viral features, host jumps and phylogeography on the rapid evolution of Aleutian mink disease virus (AMDV)
Source: Sci Rep. 2021 Aug 12;11:16464. doi: 10.1038/s41598-021-96025-z (PMC8360955; doi:10.1038/s41598-021-96025-z)
Supplement: Supplementary file 2 — Supplementary Information 2. [file 41598_2021_96025_MOESM2_ESM.pdf]

Impact of viral features, host jumps and phylogeography on the rapid evolution of Aleutian mink disease virus (AMDV).

Giovanni Franzo[1*], Matteo Legnardi[1], Laura Grassi[1], Giorgia Dotto[1], Michele Drigo[1], Mattia Cecchinato[1], Claudia Maria Tucciarone[1]

[1]Department of Animal Medicine, Production and Health (MAPS), University of Padua, 35020 Legnaro (PD), Italy;

*****Correspondence: giovanni.franzo@unipd.it

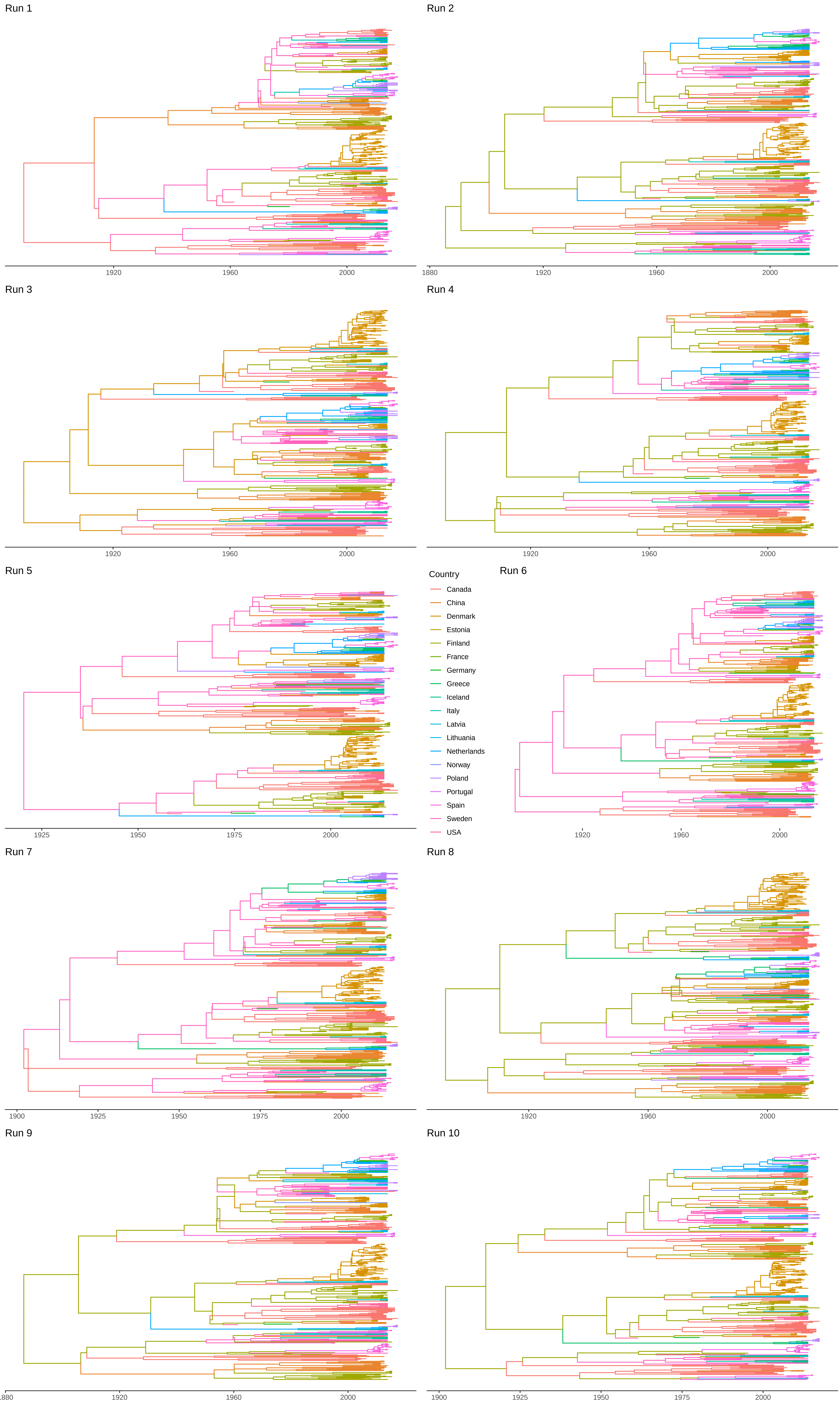
Run 1
1920
1960
2000
Run 2
1880
1920
1960
2000
Run 3
1920
1960
2000
Run 4
1920
1960
2000
Run 5
1925
1950
1975
2000
Country
Canada
China
Denmark
Estonia
Finland
France
Germany
Greece
Iceland
Italy
Latvia
Lithuania
Netherlands
Norway
Poland
Portugal
Spain
Sweden
USA
Run 6
1920
1960
2000
Run 7
1900
1925
1950
1975
2000
Run 8
1920
1960
2000
Run 9
1880
1920
1960
2000
Run 10
1900
1925
1950
1975
2000

Supplementary figure 1. Time-scaled maximum clade credibility trees obtained through independent runs based on ten randomly generated datasets. Branches have been colour-coded according to the collection country with the highest posterior probability.

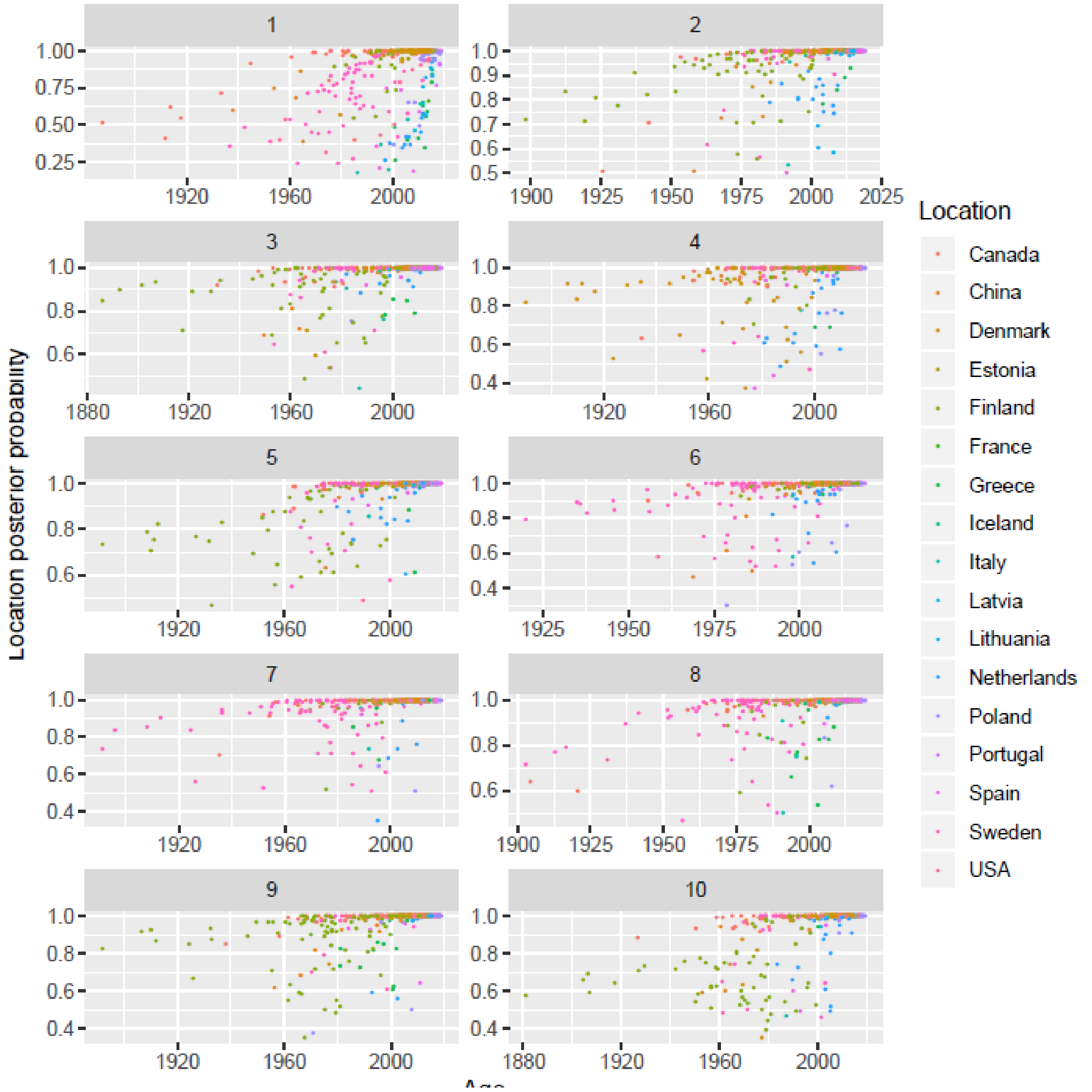

Supplementary figure 2. Scatterplot reporting the collection country posterior probability associated with the ancestral nodes. The results of the ten independent runs based on randomly generated datasets are displayed.

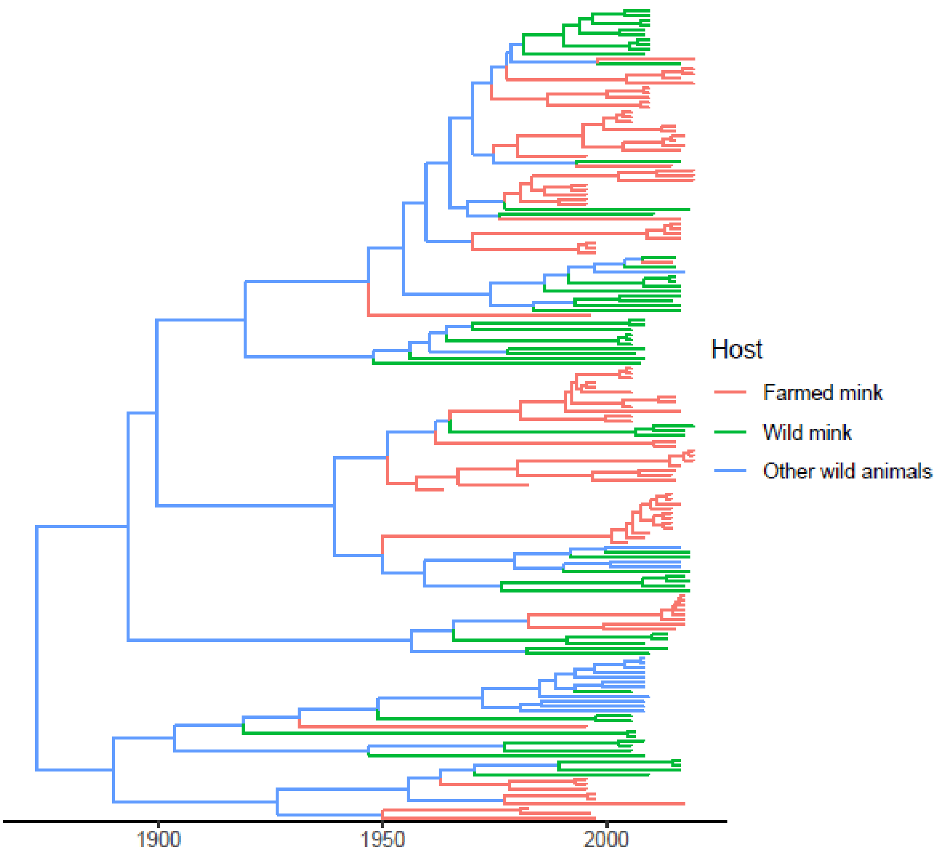

Supplementary figure 3. Time scaled maximum clade credibility phylogenetic trees obtained through the structured-coalescent based approach. Branches have been colour-coded according to the collection host with the highest posterior probability.

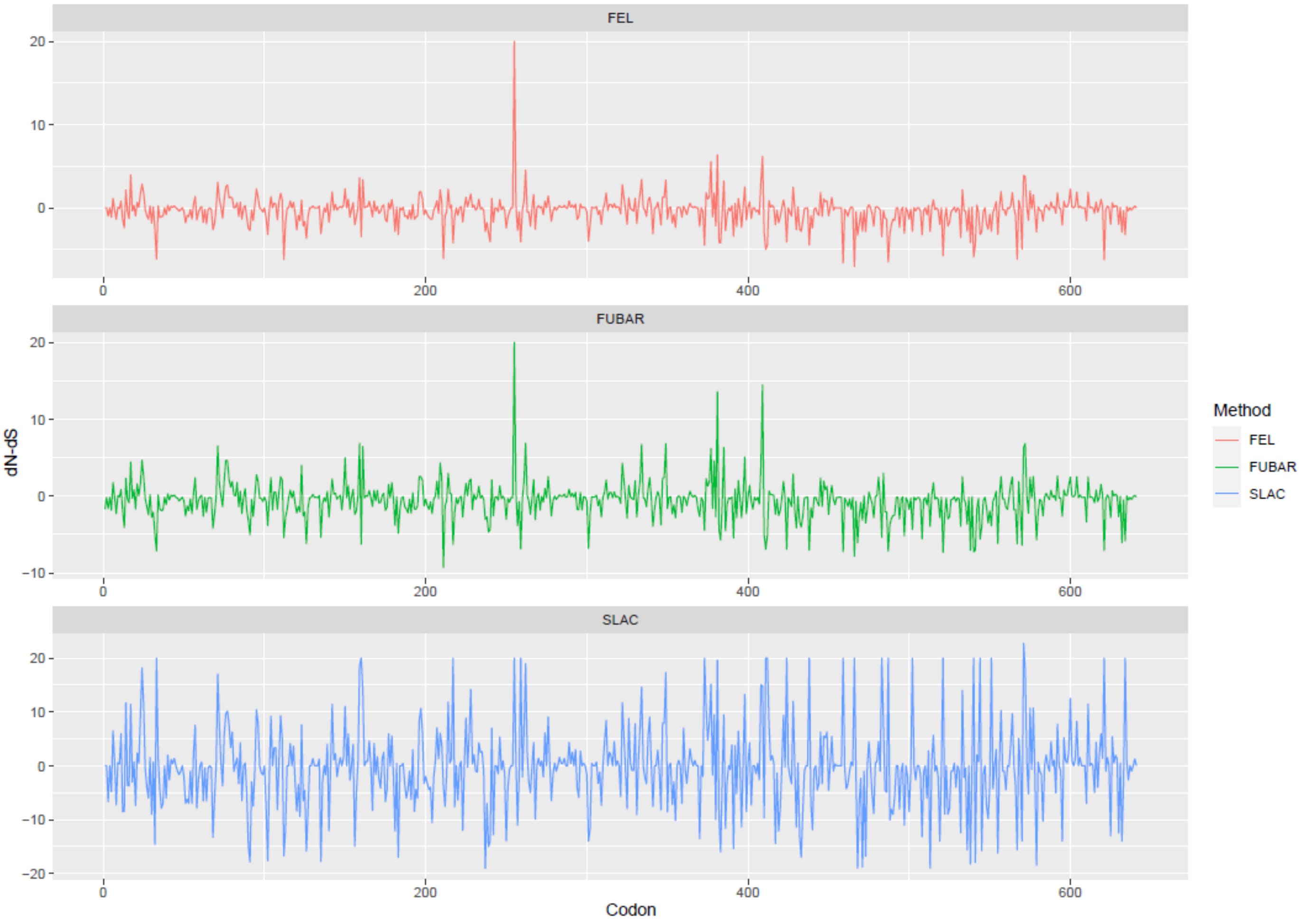

Supplementary figure 4. Plot reporting the dN-dS of NS1 codon positions calculated with different methods (FEL, FUBAR and SLAC).

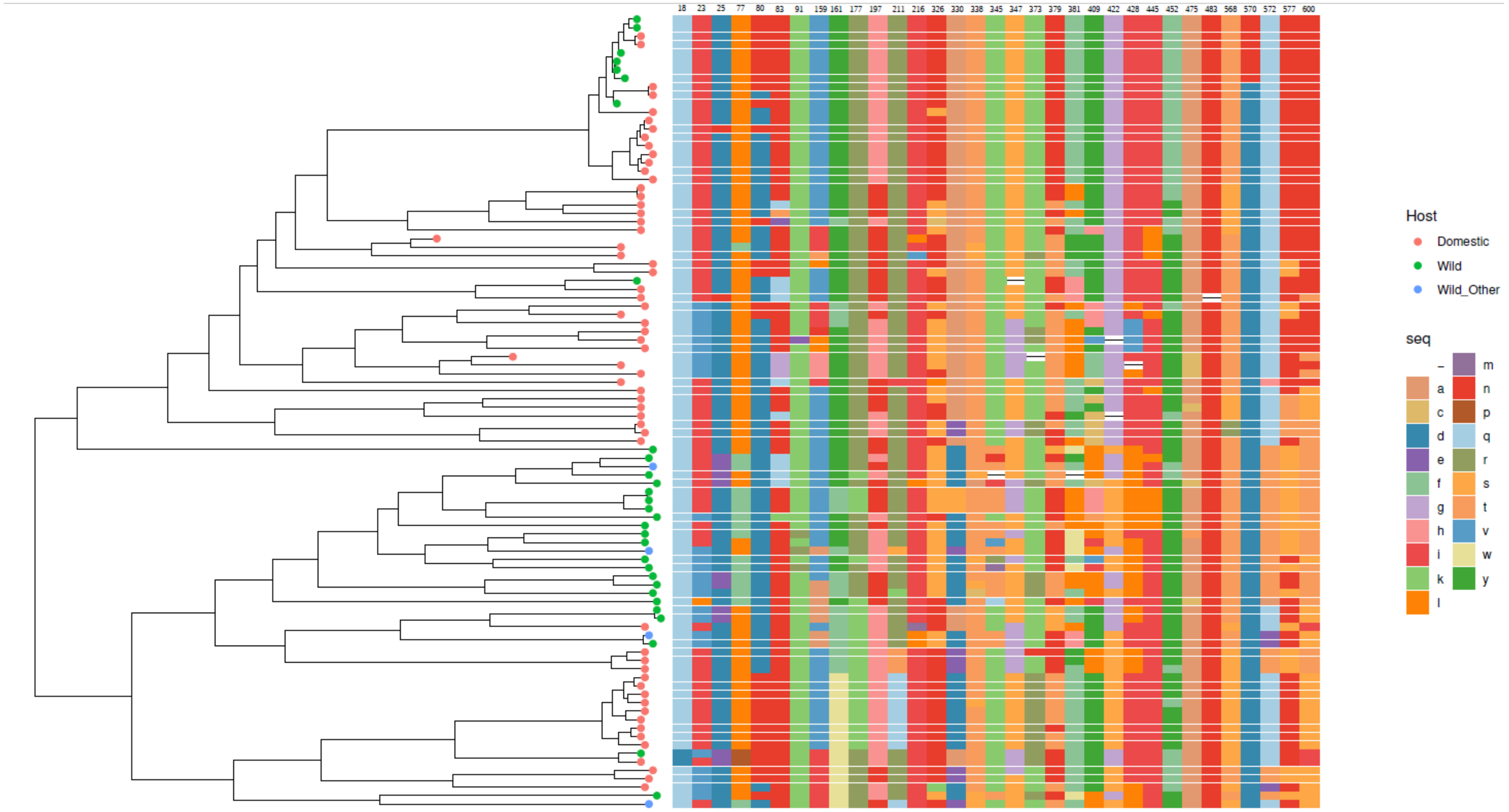

Supplementary figure 5. Plot reporting the alignment of the NS1 protein amino acids under episodic directional selection with respect to their position in the phylogenetic tree (strains have been colour-coded according to the collection host with the highest posterior probability).
