# Supplementary material for: Impact of viral features, host jumps and phylogeography on the rapid evolution of Aleutian mink disease virus (AMDV)
Source: Sci Rep. 2021 Aug 12;11:16464. doi: 10.1038/s41598-021-96025-z (PMC8360955; doi:10.1038/s41598-021-96025-z)
Supplement: Supplementary file 3 — Supplementary Information 3. [file 41598_2021_96025_MOESM3_ESM.docx]

**Impact of viral features, host jumps and phylogeography on the rapid evolution of Aleutian mink disease virus (AMDV).**

**Giovanni Franzo^1*^, Matteo Legnardi^1^, Laura Grassi^1^, Giorgia Dotto^1^, Michele Drigo^1^, Mattia Cecchinato^1^, Claudia Maria Tucciarone^1^**

^1^Department of Animal Medicine, Production and Health (MAPS), University of Padua, 35020 Legnaro (PD), Italy;

*****Correspondence: giovanni.franzo@unipd.it

|  | SLAC | | FEL | | FUBAR | |
| --- | --- | --- | --- | --- | --- | --- |
| Codon | p-value | dN-dS | p-value | dN-dS | Posterior probability | dN-dS |
| 6 | 0,006 | 6,463 | 0,007 | 1,148 | 0,992 | 1,768 |
| 11 | 0,015 | 5,971 | 0,021 | 0,813 | 0,969 | 0,851 |
| 14 | 0,019 | 11,708 | 0,004 | 2,140 | 0,983 | 2,301 |
| 17 | 0,040 | 11,409 | 0,001 | 3,963 | 0,994 | 4,423 |
| 24 | 0,000 | 18,185 | 0,007 | 2,856 | 0,998 | 4,673 |
| 25 | 0,007 | 11,195 | 0,001 | 1,809 | 0,998 | 2,508 |
| 57 | 0,002 | 7,500 | 0,003 | 1,360 | 0,998 | 2,371 |
| 71 | 0,000 | 17,017 | 0,000 | 3,056 | 1,000 | 6,536 |
| 72 | 0,002 | 7,497 | 0,007 | 1,085 | 0,991 | 1,657 |
| 76 | 0,112 | 9,657 | 0,025 | 2,507 | 0,998 | 4,643 |
| 77 | 0,005 | 10,152 | 0,004 | 2,753 | 0,972 | 4,613 |
| 78 | 0,003 | 7,631 | 0,007 | 1,223 | 0,995 | 2,189 |
| 80 | 0,035 | 6,255 | 0,038 | 1,140 | 0,978 | 1,883 |
| 95 | 0,001 | 10,379 | 0,004 | 2,275 | 0,976 | 2,748 |
| 96 | 0,010 | 7,942 | 0,059 | 1,255 | 0,938 | 1,981 |
| 104 | 0,002 | 9,212 | 0,004 | 1,350 | 0,996 | 2,413 |
| 110 | 0,001 | 9,294 | 0,001 | 1,739 | 0,998 | 2,511 |
| 116 | 0,121 | 4,117 | 0,037 | 0,814 | 0,946 | 0,773 |
| 139 | 0,039 | 4,000 | 0,051 | 0,566 | 0,928 | 0,614 |
| 142 | 0,000 | 11,423 | 0,004 | 1,918 | 0,988 | 2,237 |
| 150 | 0,005 | 11,001 | 0,016 | 2,279 | 0,937 | 4,997 |
| 152 | 0,034 | 5,917 | 0,023 | 1,033 | 0,974 | 1,409 |
| 159 | 0,000 | 18,598 | 0,000 | 3,637 | 1,000 | 6,859 |
| 161 | 0,005 | 13,988 | 0,006 | 3,399 | 0,998 | 6,453 |
| 197 | 0,011 | 10,646 | 0,000 | 1,949 | 0,999 | 2,537 |
| 207 | 0,017 | 7,040 | 0,086 | 0,862 | 0,966 | 1,939 |
| 209 | 0,207 | 6,043 | 0,039 | 2,174 | 0,994 | 4,310 |
| 214 | 0,002 | 11,827 | 0,002 | 2,240 | 0,947 | 2,965 |
| 228 | 0,000 | 14,178 | 0,008 | 1,670 | 0,986 | 2,224 |
| 241 | 0,014 | 7,000 | 0,054 | 1,142 | 0,979 | 2,124 |
| 246 | 0,067 | 5,336 | 0,006 | 0,987 | 0,992 | 1,343 |
| 261 | 0,048 | 6,323 | 0,216 | 0,820 | 0,931 | 1,874 |
| 262 | 0,000 | 18,967 | 0,000 | 4,533 | 1,000 | 6,885 |
| 274 | 0,032 | 6,124 | 0,023 | 0,788 | 0,980 | 0,978 |
| 276 | 0,020 | 9,083 | 0,002 | 1,434 | 0,999 | 2,529 |
| 289 | 0,035 | 4,320 | 0,011 | 0,855 | 0,968 | 0,894 |
| 312 | 0,018 | 8,406 | 0,036 | 1,799 | 0,940 | 2,090 |
| 322 | 0,044 | 11,731 | 0,022 | 2,801 | 0,988 | 4,305 |
| 323 | 0,032 | 5,564 | 0,018 | 1,248 | 0,986 | 2,022 |
| 330 | 0,024 | 7,796 | 0,135 | 1,154 | 0,908 | 1,978 |
| 333 | 0,118 | 7,206 | 0,025 | 1,723 | 0,917 | 1,954 |
| 334 | 0,000 | 14,606 | 0,000 | 3,423 | 1,000 | 6,731 |
| 338 | 0,013 | 5,440 | 0,018 | 0,860 | 0,973 | 0,916 |
| 339 | 0,001 | 9,023 | 0,001 | 1,289 | 0,999 | 2,394 |
| 347 | 0,024 | 7,919 | 0,046 | 1,321 | 0,978 | 2,303 |
| 348 | 0,032 | 7,931 | 0,005 | 1,263 | 0,998 | 2,417 |
| 349 | 0,000 | 17,310 | 0,000 | 3,339 | 1,000 | 6,821 |
| 374 | 0,001 | 10,886 | 0,002 | 1,827 | 0,995 | 2,280 |
| 375 | 0,027 | 4,711 | 0,006 | 1,068 | 0,980 | 1,287 |
| 376 | 0,054 | 7,869 | 0,032 | 1,273 | 0,926 | 1,854 |
| 377 | 0,031 | 15,152 | 0,004 | 5,552 | 0,992 | 6,198 |
| 379 | 0,026 | 9,467 | 0,104 | 1,790 | 0,955 | 4,573 |
| 381 | 0,001 | 19,619 | 0,000 | 6,354 | 0,999 | 13,572 |
| 385 | 0,063 | 9,445 | 0,003 | 3,239 | 0,993 | 6,317 |
| 398 | 0,002 | 13,245 | 0,004 | 2,511 | 0,970 | 5,067 |
| 409 | 0,021 | 14,788 | 0,000 | 6,196 | 1,000 | 14,467 |
| 422 | 0,006 | 9,370 | 0,137 | 0,946 | 0,917 | 1,925 |
| 428 | 0,000 | 11,988 | 0,001 | 2,494 | 0,986 | 2,866 |
| 445 | 0,088 | 6,278 | 0,008 | 1,845 | 0,978 | 2,348 |
| 448 | 0,016 | 5,140 | 0,014 | 0,732 | 0,971 | 0,803 |
| 515 | 0,031 | 5,656 | 0,042 | 0,958 | 0,976 | 1,738 |
| 533 | 0,000 | 13,979 | 0,000 | 2,199 | 0,999 | 2,528 |
| 557 | 0,001 | 10,265 | 0,000 | 1,952 | 0,999 | 2,527 |
| 564 | 0,002 | 9,694 | 0,002 | 1,833 | 0,995 | 2,472 |
| 571 | 0,000 | 22,742 | 0,000 | 3,893 | 1,000 | 6,316 |
| 572 | 0,000 | 17,571 | 0,000 | 3,755 | 1,000 | 6,828 |
| 575 | 0,002 | 10,705 | 0,001 | 2,044 | 0,993 | 2,456 |
| 577 | 0,009 | 10,719 | 0,001 | 1,572 | 0,999 | 2,541 |
| 588 | 0,026 | 4,500 | 0,029 | 0,723 | 0,957 | 0,757 |
| 592 | 0,007 | 7,698 | 0,000 | 1,814 | 1,000 | 2,624 |
| 596 | 0,040 | 5,230 | 0,055 | 0,793 | 0,952 | 0,784 |
| 600 | 0,000 | 12,497 | 0,001 | 2,245 | 0,995 | 2,478 |
| 604 | 0,002 | 8,240 | 0,000 | 1,876 | 0,999 | 2,520 |
| 611 | 0,000 | 11,462 | 0,001 | 1,873 | 0,998 | 2,505 |
| 616 | 0,158 | 4,337 | 0,030 | 0,715 | 0,969 | 0,801 |
| 619 | 0,081 | 5,914 | 0,010 | 1,018 | 0,991 | 1,504 |
| 626 | 0,015 | 5,374 | 0,047 | 0,610 | 0,943 | 0,677 |

Supplementary table 1. Codons detected under pervasive diversifying selection by at least 2 of the implemented methods. The dN-dS and the respective p-value (SLAC and FEL) or posterior probability (FUBAR) is reported for each codon and method.

| Codon | β^+^ | P^+^ | p-value | |
| --- | --- | --- | --- | --- |
| 6 | 1,140 | 0,990 | 0,010 |  |
| 11 | 0,810 | 1,000 | 0,030 |  |
| 14 | 6,840 | 0,370 | 0,000 |  |
| 17 | 45,060 | 0,200 | 0,000 |  |
| 24 | 17,560 | 0,240 | 0,000 |  |
| 25 | 1,810 | 1,000 | 0,000 |  |
| 57 | 1,390 | 0,990 | 0,010 |  |
| 64 | 43,490 | 0,050 | 0,010 |  |
| 71 | 3,290 | 1,000 | 0,000 |  |
| 72 | 4,350 | 0,280 | 0,010 |  |
| 75 | 21,610 | 0,100 | 0,000 |  |
| 76 | 15,310 | 0,320 | 0,000 |  |
| 77 | 3,480 | 0,870 | 0,010 |  |
| 78 | 3,080 | 0,420 | 0,0\10 |  |
| 79 | 121,670 | 0,020 | 0,000 |  |
| 80 | 8,010 | 0,190 | 0,030 |  |
| 83 | 49,690 | 0,060 | 0,000 |  |
| 85 | 31,080 | 0,040 | 0,000 |  |
| 88 | 29,880 | 0,050 | 0,000 |  |
| 94 | 46,060 | 0,090 | 0,000 |  |
| 95 | 37,800 | 0,100 | 0,000 |  |
| 104 | 1,340 | 1,000 | 0,010 |  |
| 107 | 35,610 | 0,020 | 0,000 |  |
| 109 | 123,840 | 0,020 | 0,000 |  |
| 110 | 1,730 | 1,000 | 0,000 |  |
| 111 | 8,240 | 0,150 | 0,010 |  |
| 142 | 7,630 | 0,300 | 0,000 |  |
| 150 | 3,050 | 1,000 | 0,020 |  |
| 152 | 1,030 | 0,990 | 0,030 |  |
| 154 | 47,690 | 0,010 | 0,000 |  |
| 159 | 10,170 | 0,390 | 0,000 |  |
| 161 | 37,450 | 0,120 | 0,000 |  |
| 168 | 30,730 | 0,040 | 0,000 |  |
| 178 | 13,440 | 0,010 | 0,040 |  |
| 192 | 35,400 | 0,020 | 0,000 |  |
| 197 | 1,950 | 1,000 | 0,000 |  |
| 209 | 23,520 | 0,160 | 0,010 |  |
| 214 | 20,810 | 0,170 | 0,000 |  |
| 215 | 27,710 | 0,150 | 0,030 |  |
| 225 | 24,190 | 0,120 | 0,000 |  |
| 228 | 7,070 | 0,280 | 0,000 |  |
| 235 | 26,900 | 0,040 | 0,000 |  |
| 246 | 9,810 | 0,120 | 0,000 |  |
| 261 | 47,170 | 0,060 | 0,000 |  |
| 262 | 16,350 | 0,320 | 0,000 |  |
| 267 | 31,030 | 0,080 | 0,000 |  |
| 274 | 0,940 | 1,000 | 0,030 |  |
| 276 | 8,340 | 0,190 | 0,000 |  |
| 289 | 0,870 | 0,990 | 0,020 |  |
| 310 | 67,130 | 0,010 | 0,000 |  |
| 314 | 10,770 | 0,050 | 0,010 |  |
| 315 | 57,170 | 0,030 | 0,000 |  |
| 322 | 21,330 | 0,330 | 0,000 |  |
| 323 | 1,470 | 1,000 | 0,030 |  |
| 333 | 2,290 | 1,000 | 0,040 |  |
| 334 | 25,290 | 0,190 | 0,000 |  |
| 338 | 9,400 | 0,130 | 0,020 |  |
| 339 | 4,210 | 0,340 | 0,000 |  |
| 345 | 15,830 | 0,130 | 0,010 |  |
| 346 | 21,300 | 0,140 | 0,010 |  |
| 348 | 2,960 | 0,450 | 0,010 |  |
| 349 | 3,330 | 1,000 | 0,000 |  |
| 361 | 46,860 | 0,010 | 0,020 |  |
| 374 | 38,410 | 0,080 | 0,000 |  |
| 375 | 1,050 | 1,000 | 0,010 |  |
| 376 | 4,020 | 0,490 | 0,040 |  |
| 377 | 19,030 | 0,370 | 0,000 |  |
| 378 | 3133,860 | 0,010 | 0,000 |  |
| 381 | 14,030 | 0,520 | 0,000 |  |
| 385 | 7,180 | 0,480 | 0,000 |  |
| 390 | 76,150 | 0,060 | 0,000 |  |
| 393 | 92,670 | 0,050 | 0,000 |  |
| 398 | 5,260 | 0,540 | 0,000 |  |
| 407 | 42,750 | 0,010 | 0,040 |  |
| 408 | 4688,000 | 0,030 | 0,000 |  |
| 409 | 44,390 | 0,220 | 0,000 |  |
| 413 | 149,150 | 0,020 | 0,000 |  |
| 428 | 2,770 | 1,000 | 0,000 |  |
| 445 | 1,950 | 0,950 | 0,010 |  |
| 448 | 18,210 | 0,070 | 0,000 |  |
| 449 | 27,440 | 0,090 | 0,000 |  |
| 450 | 92,430 | 0,050 | 0,000 |  |
| 475 | 82,230 | 0,030 | 0,000 |  |
| 480 | 12,370 | 0,010 | 0,010 |  |
| 481 | 19,550 | 0,280 | 0,010 |  |
| 484 | 32,690 | 0,120 | 0,030 |  |
| 533 | 2,270 | 0,970 | 0,000 |  |
| 548 | 11,400 | 0,120 | 0,030 |  |
| 557 | 1,950 | 1,000 | 0,000 |  |
| 563 | 64,490 | 0,080 | 0,000 |  |
| 564 | 1,850 | 1,000 | 0,000 |  |
| 571 | 9,150 | 0,500 | 0,000 |  |
| 572 | 23,680 | 0,190 | 0,000 |  |
| 575 | 9,770 | 0,260 | 0,000 |  |
| 577 | 1,590 | 1,000 | 0,000 |  |
| 588 | 0,730 | 0,990 | 0,040 |  |
| 592 | 8,990 | 0,210 | 0,000 |  |
| 600 | 2,300 | 1,000 | 0,000 |  |
| 604 | 1,890 | 1,000 | 0,000 |  |
| 611 | 35,860 | 0,090 | 0,000 |  |
| 616 | 0,720 | 1,000 | 0,040 |  |
| 618 | 167,850 | 0,030 | 0,000 |  |
| 619 | 1,020 | 1,000 | 0,020 |  |

Supplementary table 2. Codons detected under episodic diversifying selection by MEME. β^+^=Non-synonymous substitution rate at the site for the positive/neutral evolution component; p^+^ = proportion of tree evolving neutrally or under positive selecion

| **Codon** | **Directional selection toward amino acid** | **p-value** |
| --- | --- | --- |
| 18 | D | 0,001 |
| 23 | V | 0,000 |
| 25 | E | 0,001 |
| 77 | L | 0,001 |
| 80 | D | 0,002 |
| 83 | Q | 0,000 |
| 91 | R | 0,000 |
| 159 | A | 0,000 |
| 161 | F | 0,000 |
| 161 | Y | 0,001 |
| 177 | R | 0,000 |
| 197 | H | 0,000 |
| 197 | N | 0,000 |
| 211 | R | 0,000 |
| 216 | L | 0,000 |
| 326 | S | 0,001 |
| 330 | D | 0,000 |
| 338 | S | 0,001 |
| 345 | T | 0,000 |
| 347 | S | 0,000 |
| 373 | R | 0,001 |
| 379 | T | 0,000 |
| 379 | N | 0,000 |
| 381 | W | 0,000 |
| 409 | L | 0,000 |
| 422 | S | 0,001 |
| 428 | L | 0,001 |
| 445 | L | 0,001 |
| 452 | Y | 0,000 |
| 475 | C | 0,001 |
| 483 | N | 0,000 |
| 568 | T | 0,000 |
| 570 | D | 0,000 |
| 572 | T | 0,000 |
| 577 | S | 0,001 |
| 577 | N | 0,001 |
| 600 | S | 0,000 |
| 600 | T | 0,001 |

Supplementary table 3. Sites detected under episodic directional selection assuming strains collected from wild subjects as foreground branches. The amino acid toward which selection is directed is reported together with the respective p-value.
